# Enological potential and application of *Metschnikowia pulcherrima* in Vidal blanc icewine fermentation

**DOI:** 10.3389/fmicb.2026.1757951

**Published:** 2026-02-25

**Authors:** Ge Tian, Wanqing Zhang, Dafan Zhu, Jinhua Pan, Wei Qu, Yingwei Wang, Lina Zhou

**Affiliations:** 1 Department of Cardiology, The First Affiliated Hospital of Jinzhou Medical University, Jinzhou, Liaoning, China; 2 Department of Gastroenterology, The First Affiliated Hospital of Jinzhou Medical University, Jinzhou, Liaoning, China; 3 Department of Student Affairs, The First Affiliated Hospital of Jinzhou Medical University, Jinzhou, Liaoning, China; 4 The First Clinical Medical College, Jinzhou Medical University, Jinzhou, Liaoning, China; 5 Department of Geriatrics, The First Affiliated Hospital of Jinzhou Medical University, Jinzhou, Liaoning, China

**Keywords:** fermentation property, icewine, *Metschnikowia pulcherrima*, mixed culture fermentation, volatile aroma compounds

## Abstract

Metschnikowia pulcherrima has been studied and applied in winemaking in recent years, but its application in icewine fermentation has rarely been reported. In this study, the tolerance and β-glucosidase activities of indigenous M. pulcherrima strain were first detected. The results show that, except for a relatively low tolerance to ethanol [limited to 4% (v/v)], the other tolerance is good to the fermentation of icewine; the cell wall-bound and extracellular β-glucosidase activities of *M. pulcherrima* strain were 35.93 nmol/mL (1.28 U) and 14.31 nmol/mL (0.42 U), respectively. M. pulcherrima and *Saccharomyces cerevisiae* (commercial and indigenous) strains were sequentially inoculated for icewine fermentations; meanwhile, pure S. cerevisiae fermentations were used as the control. The results showed that M. pulcherrima was completely replaced by S. cerevisiae in the middle and late stages of mixed culture fermentations of icewine. Compared with the icewine fermented with pure S. cerevisiae, the concentration of acetic acid and ethanol was lower, and the concentration of glycerol was higher in the mixed culture fermented icewines. After inoculation with M. pulcherrima, the levels of several important volatile compounds increased significantly, including β-damascenone, trans-rose oxide, isobutanol, isoamyl acetate, isobutyl acetate, and some ethyl esters (ethyl acetate, ethyl caprylate, ethyl caprate, ethyl nonanoate, ethyl hexanoate, and ethyl 9-decenoate). The pleasant fruity and flowery characteristics of mixed-fermentation icewine was intensified.

## 1 Introduction

Icewine is a naturally sweet wine made from frozen grapes that remain on the vine in the cold winter weather, and these frozen grapes are harvested and squeezed at a natural temperature of ≤−7°C (OIV, 2018). Concentrated grape must contains high concentrations of sugars (soluble solids level > 35*^o^*Bx), acids and aromatic compounds for further production of icewine, while most of the water in frozen grapes is removed in the form of ice crystal along with the frozen grape skins (Li et al., 2018). Due to the demanding environmental and climatic requirements of ice grape growth, icewine is mainly produced in a few countries such as Canada, Germany, Austria, and China. In recent years, China’s icewine industry has experienced rapid growth, with an annual production of approximately 3 million liters (Lan et al., 2019). This expansion highlights the need for innovative fermentation strategies to enhance product differentiation in the global market. Vidal blanc is a white grape cultivar widely used in the production of icewine because of its high resistance to harsh climates and relatively stable yield, and its icewine has an appealing aroma and charming flavor (Ma et al., 2017; Crandles et al., 2015).

In the process of wine fermentation, the microbiota changes dynamically, in which yeast plays an important role, and a dynamic succession of yeast communities occurs (Ballester-Tomás et al., 2017). *Saccharomyces* and non-*Saccharomyces* yeasts work together to convert grape juice into wine and determine the sensory quality of the wine (Li et al., 2018). During the fermentation of icewine, initially many aroma substances (such as musk, rose, lychee, etc.) do not exist in a free state but rather in an odorless and tasteless glycoside form (bound to a glucose molecule) in ice grape juicy. The content of combined-state aroma glycosides in grape berries is 2–8 times that of free-state aroma substances (Liu et al., 2017), and 90% of the sugar compounds are hydrolyzed by sugar hydrolases produced by yeast. Some studies have found that non-*Saccharomyces* yeasts used for winemaking can secrete several beneficial hydrolytic enzymes, among which the most notable is β-glucosidase (Zhang et al., 2020), and *M. pulcherrima* is one of the excellent producers of β-glucosidase among non-*Saccharomyces* yeasts (Zhang et al., 2021). β-Glucosidase predominantly targets the β-1,4-glycosidic bonds, hydrolyzing the bound flavor precursors into free volatile aroma substances, which is a key enzyme that affects the flavor and taste quality of wine. Therefore, non-*Saccharomyces* yeasts can play a role in increasing the level of aroma compounds, such as terpenes and C13-norisoprenoids (Hong et al., 2019; Tronchoni et al., 2017).

Metschnikowia pulcherrima, whose name means “the most beautiful” yeast in Latin, is a non-*Saccharomyces* species that has been studied and focused on in recent years. It has been found in grape must and could co-exist with a commercial *S. cerevisiae* strain during fermentation (Mandakovic et al., 2020). It has been used in wine-making, and has significant potential for producing wines with low ethanol content, high acetate and higher alcohols (Hranilovic et al., 2018). Moreover, it has also been found in the spontaneous fermentation of some sweet wine such as icewine and botrytized wines, and its presence can be detected even in the late stages of fermentation (Bokulich et al., 2012; Li et al., 2018). But, there are very few research about the application of *M. pulcherrima* in sweet wine fermentation.

Recently, the use of indigenous yeasts such as *Starmerella bacillaris*, *Hanseniaspora uvarum*, *Torulaspora delbrueckii* to increase the complexity of wine aroma has gradually become a trend of winemaking with regional characteristics (Li et al., 2022; Hong et al., 2021). The indigenous *M. pulcherrima* strains were found and isolated in the early and middle stages of spontaneous fermentation of Vidal blanc icewine. This study mainly investigated the fermentation characteristics of *M. pulcherrima* strains and the dynamic changes during icewine fermentation process, as well as the effects on aromatic profile of Vidal blanc icewine. The main objective was to demonstrate that co-fermentation of *M. pulcherrima* and *S. cerevisiae* can provide the distinct and complex aromatic profile of icewine while maintaining fermentation efficiency.

## 2 Materials and methods

### 2.1 Yeast strains

The two yeast strains used in this study (*M. pulcherrima* and *S. cerevisiae*) were isolated from the spontaneous fermentation of Vidal blanc icewine, and were identified using Wallerstein laboratory nutrient (WLN) agar medium and the internal transcribed spacer (ITS) region sequences method (Li et al., 2018). The strain of *M. pulcherrima* was numbered MF1, and the strain of *S. cerevisiae* was designated S2. The commercial yeast (ST^§^, LAFFORT, Bordeaux, France) was used and designated S1.

### 2.2 Tolerance properties of yeast strains

The strains of *M. pulcherrima* and *S. cerevisiae* were activated in YPD (yeast extract 10 g/L, peptone 20 g/L, dextrose 20 g/L, all from Haibo, Qingdao, China) medium at 28°C for 48 h. Then, a series of tolerance experiments (sugar, tartaric acid, alcohol and SO_2_ concentrations) were carried out using Durham’s fermentation tube method, and the presence of viable yeast cells was checked using YPD agar medium. The tolerance medium was based on YPD medium, the tolerance gradients were as follow: glucose concentrations were 300, 350, 400, 450, and 500 (g/L); tartaric acid concentrations were 4, 8, 12, 16, and 20 (g/L); ethanol concentrations were 4, 8, 10, 12, and 14 (%, v/v); and total amount of SO_2_ concentrations were 100, 150, 200, 250, 300, and 350 (mg/L). The tolerance trials were conducted using the gradients described by Hong et al. (2019).

### 2.3 β-glucosidase activity of *M. pulcherrima* strain

The strains of *M. pulcherrima* was inoculated in YPD medium, and cultured at 28 °C for 24 h (OD600 ≈ 1.0). After centrifugation at 11,000 × *g* for 10 min, cells were collected and inoculated in the induction medium (YNB medium supplemented with 10 g/L glucose and 2% xylan) with a concentration of 10^6^ cells/mL, and incubated at 28°C for 48 h. After another centrifugation, both the cells and the supernatant were collected for further analysis, and enzyme activities of cell wall-bound and extracellular enzymes were measured. The β-glucosidase activities were determined by quantifying the amount of pNP released from 4-nitrophenyl-β-D-glucopyranoside (Yuanye, Shanghai, China), which was used as the substrate. The measurements were compared against a series of standard solutions prepared with pNP at concentrations ranging from 10 to 60 nmol/mL. The reaction mixtures contained 0.75 mL of supernatant (or the yeasts were washed and resuspended in 0.75 mL of 0.2 M citric-0.1 M phosphate buffer, pH 5.0) and 0.25 mL of 5 mM pNP-substrate in the same buffer was incubated at 40 °C for 90 min. The reaction was terminated by adding 1.0 mL of 0.2 M Na_2_CO_3_, and the absorption value was measured at 404 nm after standing. One unit of β-glucosidase activity was defined as the quantity of enzyme required to release 1 nmol of pNP per hour under the specified assay conditions (Hong et al., 2019).

### 2.4 Fermentation trials

The raw ice grape juice used for icewine fermentation had a pH of 4.03, soluble solid content of 41.0°Bx, and total acid and sugar contents of 4.98 and 432.97 g/L, respectively. Each 180 mL of the ice grape juice was added with 50 mg/L SO_2_ and placed in 250 mL sterile flask with sterile glass air-lock (containing concentrated sulfuric acid), and then each flask was heated at 70°C for 20 min to sterilize (Li and Hong, 2023; Hong et al., 2021). The commercial *S. cerevisiae* (S1) was activated, and the indigenous strains of *M. pulcherrima* (MF1) and *S. cerevisiae* (S2) were activated in YPD medium. The fermentations of laboratory scale for this study were performed as follows: (1) monoculture fermentation of S1; (2) monoculture fermentation of S2; (3) mixed culture fermentation: sequential inoculation of MF1 followed by S1 after 48 h (named as MF1S1); (4) mixed culture fermentation: sequential inoculation of MF1 followed by S2 after 48 h (named as MF1S2). The initial fermentation concentration of *M. pulcherrima* and *S. cerevisiae* were about 10^6^ cells/mL, and the addition ratio was approximately 1: 1. All fermentation trials were carried out at 18 °C for 30 days in constant conditions (Li and Hong, 2023).

### 2.5 Yeast population dynamics during fermentation

Samples were collected at 0, 2, 4, 7, 14, 21, and 30 days during fermentation to monitor yeast population dynamics, and all the samples were analyzed in triplicate. The samples were firstly serially diluted (1, 10^4^ to 1, 10^6^ ratios) with sterile physiological solution, and then spread-plated on WLN agar medium (Haibo, Qingdao, China). All plates were incubated at 28°C for 5 days. The colonies of *M. pulcherrima* and *S. cerevisiae* could be differentiated on WLN agar medium, and plates with 20–100 colonies were selected and counted and recorded. WLN agar medium is able to monitor the changes in the yeast population, based on the color and morphology of the colonies (Alessandria et al., 2013; Li et al., 2018).

### 2.6 Basic chemical compositions

The fermentation flasks were weighed daily to record the daily CO_2_ production under different fermentation strategies. Total sugars and total acids were measured according to the inspection standards proposed by the OIV (2018). Ethanol content was analyzed using a Gas Chromatography system (GC 9790 Plus) with a flame ionization detector (Fuli Analytical Instruments Co., Zhejiang, China). The chromatograph was equipped with a KB-5 capillary column (30 m × 320 μm × 0.25 μm; Kromat Co., Bordentown, United States). Ethanol was used as the standard for apparatus calibrating, and 1-propanol was used as the internal standard, both of which were GC standard reagents (Aladdin Biochemical Technology Co., Shanghai, China). The temperature of the column oven was initially held at 45 °C for 5 min, then increased by 5 °C/min to 50 °C for 5 min, and finally increased to 230 °C at 20 °C/min for 2 min. The temperatures of injector and detector were both 250 °C. The carrier gas was Nitrogen (99.999%), the flow-rate was 1 mL/min, the injection split ratio was 50: 1, and the injection volume was 1 μL. Main organic acid (tartaric acid and acetic acid) and glycerol contents were determined by using a Prominence LC-20A system (Shimadzu Co., Japan) for high performance liquid chromatography (HPLC). The chromatograph was equipped with a Wondasil C18-WR column (4.6 mm × 150 mm, 5 μm; Shimadzu Co., Japan). The column temperature was 35 °C, the mobile phase was acetonitrile-phosphoric acid (pH 2.0) with a ratio of 2: 98, and the flow rate was 0.8 mL/min. Every basic chemical composition of each icewine sample was determined in triplicate.

### 2.7 Volatile aroma compounds

Volatile aroma compounds of the icewines fermented by different strategies were determined by HeadSpace Solid Phase MicroExtraction Gas Chromatography Tandem Time-of-Flight Mass Spectrometry (HS-SPME-GC-TOFMS), and each icewine was measured six times. A 5 mL sample of icewine and 1 g NaCl were put into a 15 mL microextraction vial and then mixed with 10 μL of 4-methyl-2-pentanol (as internal standard substance, 1.0018 g/L). The vial with a PTFE-silicon septum cover was equilibrated at 40 °C for 30 min on a heated magnetic stirrer with agitation at 300 rpm. Headspace solid-phase micro-extraction (HS-SPME) was coupled with DVB/CAR/PDMS 50/30 μm SPME fiber (Supperco, belfonte, PA, USA) and then processed by gas chromatography-mass spectrometry (GC-MS-QP2010PLUS, Shimadzu, Kyoto, Japan) to detect volatile compounds. The thermal desorption of SPME fiber in the GC injector was performed for 8 min. A Rxi™-5ms capillary column (30 m × 0.25 mm × 0.25 μm; J&W Scientific, Folsom, CA, United States) was used, and helium (99.999%) was regarded as the carrier gas and flowed with the rate of 1.0 ml/min. Injections were in split mode at 10: 1. The temperatures of the injection port, interface and ion source were 250 °C, 230 °C, and 200 °C, respectively. The column oven temperature was gradient heating and the details was as follows: the initial temperature was 35 °C (for 3 min), then raised to 160°C at a rate of 6°C/min, and finally raised to 250 °C at a rate of 10 °C/min. The mass spectrometer was performed in electron ionization (EI) mode at 70 ev with the full scan mode (m/z 35–350). Identification of volatile compounds was based on a comparison of the mass spectrum (MS) matching in the NIST05 standard library (compounds with a retention matching degree of ≥80%) and the retention indices reported in the GCMS solutions (version 2.6). Analyses were carried out in triplicate.

### 2.8 Statistical analysis

The statistical analyses were used with SPSS version 17.0 Statistical Package for Windows (SPSS Inc., USA). One-way analysis of variance (ANOVA) and Duncan’s test (p < 0.05) were used to compare the differences in the chemical components of the icewines by different inoculation trials, and the results are expressed as the mean ± SD of triplicates. Peak area intensity data for volatile aroma compounds were normalized by “Autoscaling” (mean-centered/standard deviation of each variable) and MetaboAnalyst 2.0^1^ was used to perform principal component analysis (PCA) and hierarchical cluster heatmapping through section “2.8 Statistical analysis,” as reported by Lan et al. (2016).

## 3 Results

### 3.1 Tolerance performance of yeast strains

The tolerance performances of *M. pulcherrima* and *S. cerevisiae* strains are shown in Table 1. The range of the tolerance concentration gradients was determined based on the characteristics of the ice grape juice and icewine (sugar, acidity, alcohol content) and the allowable limit of production indicators (total amount of SO_2_). The two indigenous strains were isolated and screened from the spontaneous fermentation of icewine, and the tolerance results of sugar (300, 350, 400, 450, and 500 g/L), tartaric acid (4, 8, 12, 16, and 20 g/L) and total amount of SO_2_ (100, 150, 200, 250, and 300 mg/L) were all positive. The ethanol tolerance of the *M. pulcherrima* strain was limited to 4% (v/v), whereas the *S. cerevisiae* strain tolerated up to 12% (v/v).

**TABLE 1:** Tolerance results of the indigenous *M. pulcherrima* and *S. cerevisiae* strains.

Species	Glucose (g/L)					Tartaric acid (g/L)					Ethanol (%)					SO_2_ (mg/L)				
	300	350	400	450	500	4	8	12	16	20	4	8	10	12	14	100	150	200	250	300
***M. pulcherrima***	+	+	+	+	+	+	+	+	+	+	+	–	–	–	–	+	+	+	+	+
***S. cerevisiae***	+	+	+	+	+	+	+	+	+	+	+	+	+	+	–	+	+	+	+	+

### 3.2 β-Glucosidase activity analysis of *M. pulcherrima* strain

β-Glucosidase activity can substantially influence the release and composition of aroma compounds in wine (Fernández-Pacheco et al., 2021). The results of the assays for cell wall-bound and extracellular β-glucosidase activities of *M. pulcherrima* strain were as follows: the former was 35.93 nmol/mL (1.28 U), and the latter was 14.31 nmol/mL (0.42 U). Generally, the β-glucosidase activity of non-*Saccharomyces* yeast involved in wine making is higher than that of *S. cerevisiae* (Vernocchi et al., 2015).

### 3.3 Dynamic changes of yeast growth during fermentation of icewines

**FIGURE 1:**
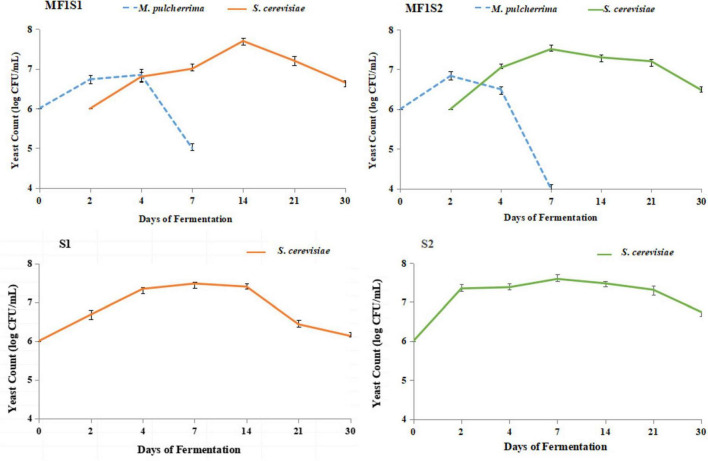
Dynamic changes of *M. pulcherrima* and *S. cerevisiae* during fermentation process.

The dynamics of *M. pulcherrima* and *S. cerevisiae* during the fermentation process, as detected by plate counts, are illustrated in Figure 1. During monoculture fermentations of *S. cerevisiae*, the cell growth trend initially increased and then decreased. The maximum cell population appeared earlier in S2 than in S1. Specifically, the cell population in S1 peaked on day 14 (7.48 log CFU/mL), while that in S2 reached its maximum on day 7 (7.59 log CFU/mL). By day 30, *S. cerevisiae* still exhibited good vitality, with cell concentrations of approximately 6.13 log CFU/mL in S1 and 6.75 log CFU/mL in S2.

During mixed culture fermentations of MF1S1, the cellular concentration of *M. pulcherrima* first increased, reached the maximum value (6.85 log CFU/ml) on day 4, and then decreased, which was undetectable in the latter half of the fermentation period; the cell population of *S. cerevisiae* gradually increased, peaking on day 14 (7.7 log CFU/mL), and subsequently declined. In MF1S2, the cell growth trend of *M. pulcherrima* similar to that observed in MF1S1, except that the maximum value occurred on day 2 (6.84 log CFU/ml) and it was undetectable after day 7; the maximum cell concentration of *S. cerevisiae* in MF1S2 was 7.51 log CFU/ml on day 7.

### 3.4 Basic chemical compositions in icewines

The basic chemical parameters of monoculture and mixed culture fermented icewines are shown in Table 2, including residual sugar, acetic acid, titratable acidity, glycerol and ethanol. Residual sugar concentration of all icewines ranged from 209.58 ± 1.10 g/L (S1) to 231 ± 2.13 g/L (MFS2). The concentrations of acetic acid in mixed culture fermented icewines were lower than those of monoculture fermented icewines, the minimum value is 1.72 ± 0.02 g/L (MFS2), and the maximum value is 1.98 ± 0.03 g/L (S1), both of which were lower than the maximum allowable limit (2.1 g/L) stipulated in OIV standard. Titratable acidity concentration ranged from 5.31 ± 0.45 g/L (MFS2) to 5.95 ± 0.25 g/L (S1). Glycerol concentration ranged from 10.67 ± 0.38 g/L (S1) to 11.78 ± 0.05 g/L (MFS2); compared with the control fermentations, the mixed culture fermentation produced a higher glycerol contents. Moreover, MFS2 icewine had the lowest ethanol concentration (11.54% ± 0.20%) and S1 icewine with single inoculation of had the highest ethanol concentration (12.57% ± 0.25%); the ethanol concentrations of mixed culture fermentations with *M. pulcherrima* and *S. cerevisiae* were lower than those of the control fermentations. The total CO_2_ productions by the control fermentations are higher than those of mixed culture fermentation (Supplementary Figure 1).

**TABLE 2:** Basic parameters of the final icewines.

Name	Residual sugar (g/L)	Acetic acid (g/L)	Titratable acidity (g/L)	Glycerol (g/L)	Ethanol (%, v/v)
S1	209.58 ± 1.10^a^	1.98 ± 0.03^d^	5.95 ± 0.25^a^	10.67 ± 0.38^b^	12.57 ± 0.25^a^
MFS1	217.46 ± 1.24^c^	1.75 ± 0.01^b^	5.78 ± 0.77^d^	11.25 ± 0.09^a^	11.97 ± 0.11^a^
S2	219.88 ± 1.22^a^	1.79 ± 0.01^a^	5.74 ± 0.37^c^	10.98 ± 0.41^c^	11.75 ± 0.05^b^
MFS2	231 ± 2.13^b^	1.72 ± 0.02^c^	5.31 ± 0.45^b^	11.78 ± 0.05^a^	11.54 ± 0.20^c^

Values are means ± standard deviations (*n* = 3). Different letters in the same column indicate significant differences (*p* < 0.05).

### 3.5 Aroma compounds analysis

**FIGURE 2:**
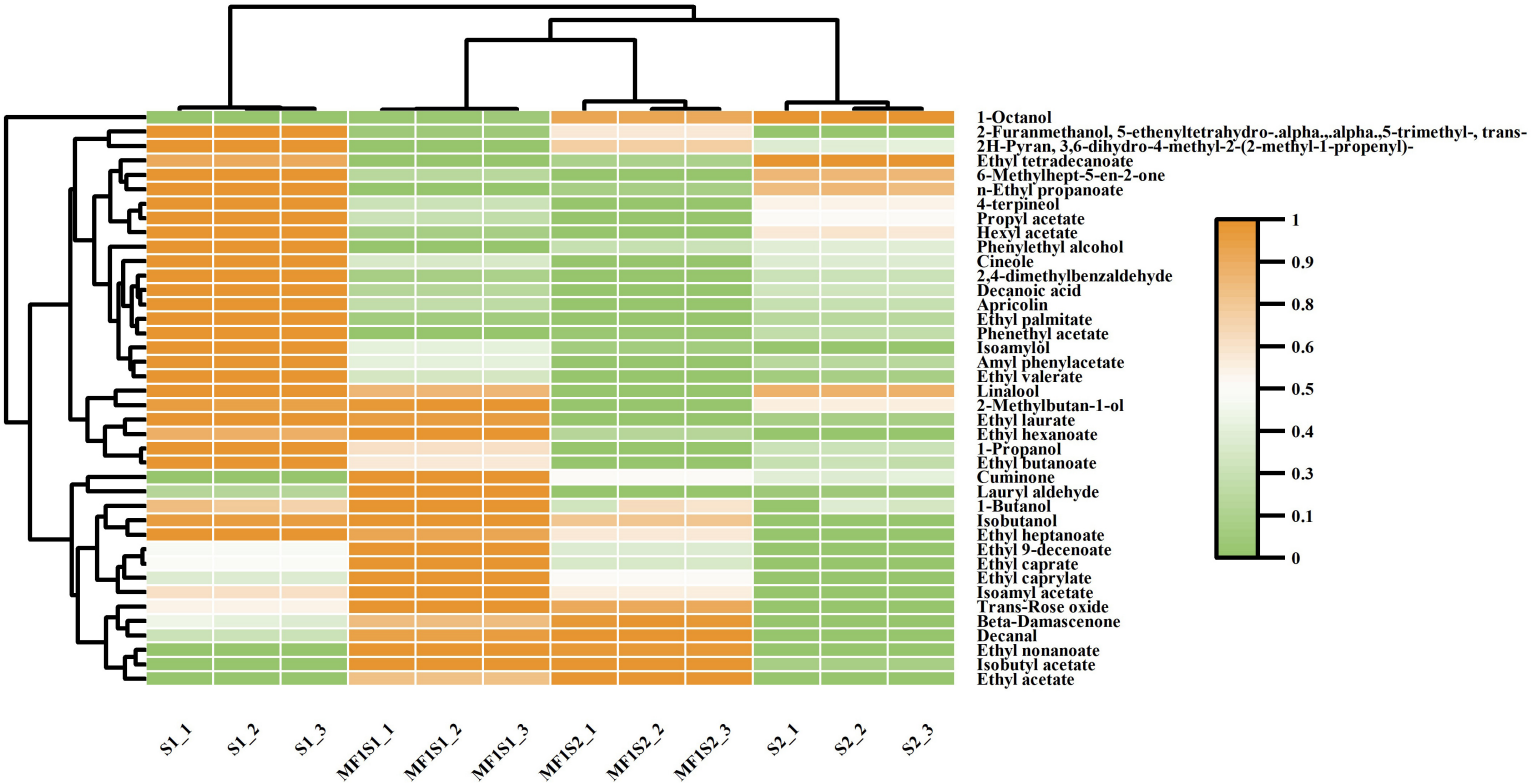
Hierarchial clustering and heatmap visualization of volatile aroma compounds in icewines produced by monoculture and mixed culture fermentation.

The volatile aroma compounds of icewines fermented separately in monoculture and mixed culture fermentations were analyzed using HS-SPME-GC-MS. A total of 40 major volatile aroma compounds were identified, including 7 higher alcohols, 20 esters, 4 aldehydes and acids, 9 terpenes and others (Figure 2). Their aroma descriptors are showed in Table 3.

**TABLE 3:** Volatile aroma compounds identified and their aroma descriptors in this study.

Compounds	CAS	RI^a^	Aroma descriptor	Reference of aroma descriptor
**Alcohols (7)**				
Isoamylol	123-51-3	697	Apple, nail polish, alcohol	Li et al., 2022
Isobutanol	78-83-1	597	Alcohol, solvent, green, bitter	Cai et al., 2014
1-Propanol	71-23-8	597	Alcohol, ripe fruit	Cai et al., 2014
1-Octanol	111-87-5	1059	Jasmine, lemon	Cai et al., 2014
1-Butanol	71-36-3	697	Fruity, floral	Cai et al., 2014
Phenylethyl alcohol	60-12-8	1136	Flowery, roses, honey	Cai et al., 2014
2-Methylbutan-1-ol	137-32-6	697	Cheese, sweet	Li et al., 2022
**Esters (20)**				
Isoamyl acetate	123-92-2	820	Banana, fruity, sweet	Li et al., 2022
Isobutyl acetate	110-19-0	721	Flowery, fruity	Hong et al., 2021
Hexyl acetate	142-92-7	984	Apple, cherry, pear, floral	Hong et al., 2021
Propyl acetate	109-60-4	686	Celery	Peinado et al., 2006
Phenethyl acetate	103-45-7	1259	Fruity, rose	Peinado et al., 2006
Ethyl hexanoate	123-66-0	984	Banana, green apple	Cai et al., 2014
Ethyl laurate	106-33-2	1580	Fruity, creamy, sweet, floral	Li et al., 2022
Ethyl butanoate	105-54-4	785	Banana, pineapple, strawberry	Cai et al., 2014
Ethyl valerate	539-82-2	884	Sweet, strawberry, apple, pineapple	Li et al., 2022
Ethyl acetate	141-78-6	586	Pineapple, fruity, solvent	Peinado et al., 2006
Ethyl palmitate	628-97-7	1978	Fruity	Li et al., 2022
Ethyl tetradecanoate	124-06-1	1779	Coconut, iris, sweet beeswax aromas	Li et al., 2022
Ethyl caprylate	106-32-1	1381	Sweet, fruity, flowery	Li et al., 2022
Ethyl caprate	110-38-3	1381	Fruity	Li et al., 2022
Ethyl 9-decenoate	67233-91-4	1371	/	Hong et al., 2021
Ethyl heptanoate	106-30-9	1083	Cherry, grape, pineapple	Cai et al., 2014
Amyl phenylacetate	5137-52-0	/	Fruity, honey, musk, cocoa	/
Apricolin	104-61-0	/	Coconut, peach	Wu et al., 2024
Ethyl nonanoate	123-29-5	1282	Fruity, rose	Li et al., 2022
n-Ethyl propanoate	105-37-3	/	Fruity, flowery	/
**Aldehydes and acids (4)**				
2,4-Dimethylbenzaldehyde	15764-16-6	1208	Cherry, almond, vanilla	Li et al., 2022
Lauryl aldehyde	112-54-9	/	Citrus	Cai et al., 2014
Decanal	112-31-2	1204	Grassy, intense citrus	Li et al., 2022
Decanoic acid	334-48-5	1372	Citrus, rancid sour	Li et al., 2022
**Terpenes, C13-norisoprenoids and others (7)**				
Linalool	78-70-6	1082	Flowery, muscat	Cai et al., 2014
4-Terpineol	562-74-3	/	Nutmeg, wood fragrance	/
Beta-Damascenone	23726-93-4	1440	Sweet, honey, exotic flowers, rose, Fruity (apple, grape, blueberry)	Cai et al., 2014
Trans-Rose oxide	876-18-6	1114	Lychee, green, rose	Li et al., 2022
Cuminone	645-13-6	1277	Spicy, woody, herbaceous	Hong et al., 2021
Cineole	470-82-6	1059	Pine, camphor, pungent, lavender oil	Li et al., 2022
6-Methylhept-5-en-2-one	110-93-0	/	Lemon-grass, fruity	Radonić et al., 2019

*^a^*Retention indices on Rxi™-5ms column.

#### 3.5.1 Higher alcohols

Seven higher alcohols detected were isoamylol, isobutanol, 1-propanol, 1-butanol, 1-octanol, phenylethyl alcohol, and 2-methylbutan-1-ol. The levels of phenylethyl alcohol and 1-propanol in the monoculture fermentations were higher than those in the mixed culture fermentations; the levels of isobutanol and 1-butanol were elevated in the icewines of mixed culture fermentations compared to the icewines of monoculture fermentations. The level of isoamylol in S1 was significantly higher than that in MF1S1, whereas it was higher in MF1S2 compared to S2; conversely, the levels of 1-octanol and 2-methylbutan-1-ol in MF1S1 were significantly higher than those in S1, while the levels of these two alcohols in S2 were both higher than those in MF1S2.

#### 3.5.2 Esters

Esters compounds are another important type of compounds in icewine, with a total of twenty ester compounds detected. The levels of isoamyl acetate and isobutyl acetate in mixed culture fermentation were significantly higher than those in monoculture fermentation, while the levels of propyl acetate and phenylethyl acetate in monoculture fermentation were significantly higher than those in mixed culture fermentation. Moreover, *M. pulcherrima* could reduce the levels of apricolin, amyl phenylacetate and hexyl acetate.

In term of ethyl esters, the levels of ethyl butanoate, ethyl laurate, ethyl tetradecanoa, ethyl valerate, ethyl palmitate, and n-ethyl propanoate in monoculture fermentation were significantly higher than those in mixed culture fermentation. In contrast, the levels of ethyl acetate, ethyl caprylate, ethyl caprate, ethyl nonanoate, ethyl hexanoate, and ethyl 9-decenoate were higher in the mixed culture fermented icewine. Besides, the levels of ethyl heptanoate in S1 were higher than those in MF1S1, while the levels in S2 were lower than those in MF1S2.

#### 3.5.3 Aldehydes and acids

There are one acid and three aldehydes were identified in this study. The levels of decanoic acid (a medium-chain fatty acids) in MF1S1 were significantly lower than those in the S1; however, it was detected to be present in S2, but it was absent in MF1S2. Moreover, the levels of 2, 4-dimethylbenzaldehyde in mixed fermented icewines were significantly lower compared to those in monoculture fermented icewines. The levels of lauryl aldehyde in MF1S1 were significantly higher than those in the S1, while the levels in MF1S2 were lower than those in S2. Besides, the levels of decanal in mixed culture fermentation were significantly higher than those in monoculture fermentation.

#### 3.5.4 Terpenes, C13-norisoprenoids and others

Terpenes are the largest category of volatile substances in the aroma compounds of wine varieties. Terpenes and C13-norisoprenoids have an important effect on the aroma of icewine, adding pleasant floral fragrance and fruity scent to it. β-glucosidase can hydrolyze glycoside substances, releasing free volatile compounds, such as monoterpenols and C-13 norisoprenoid compounds (Hong et al., 2021). The levels of β-damascenone and trans-rose oxide in mixed fermented icewines were significantly higher than those in monoculture fermented icewines. The levels of linalool and 4-terpineol were highest in S1 fermentation, followed by S2, which indicated that *M. pulcherrima* could reduce these two terpenes.

## 4 Discussion

In this study, we demonstrated the potential of the indigenous *M. pulcherrima* and *S. cerevisiae* strains for fermenting icewine, including the tolerance performance and β-glucosidase activity of the strains, and their dynamic changes in growth during the fermentation process. Firstly, the two strains, *M. pulcherrima* and *S. cerevisiae*, were isolated from the spontaneous fermentation of icewine; the total sugar and tartaric acid contents of raw ice grape juice used for the spontaneous fermentation were 483 and 11.9 g/L respectively, and the residual sugar and ethanol content of final icewine were 347 g/L and 8.0% (v/v) respectively. Their ability to tolerate high levels of sugar, acid and SO_2_, is due to the fact that these strains may have evolved to be highly responsive and adaptive to changes in the environment (Hong et al., 2019). *M. pulcherrima* strain used in this study was isolated from the early stage of spontaneous fermentation, this species was absent in the later stage, the ethanol tolerance of this *M. pulcherrima* strain should be lower than 8% (v/v); however, *S. cerevisiae* strain used in this study was obtained from at the end stage of the fermentation, so the ethanol tolerance of this *S. cerevisiae* strain should be greater than 8% (v/v). The results of ethanol tolerance of the two strains in this study were in line with expectations. Moreover, the β-glucosidase activities of non-*Saccharomyces* yeasts during wine fermentation is generally higher than that of *S. cerevisiae*. In this study, β-glucosidase activity of *S. cerevisiae* was detected through the preliminary experiment, and the result was close to 0. The research results of Krêgiel et al. (2022) also indicated that β-glucosidase activity of *S. cerevisiae* was also 0, *M. pulcherrima* was able to expressing greater β-glucosidase activities, but there were differences among different strains (Morata et al., 2019).

In terms of the dynamic changes in cell growth during the icewine fermentation process, whether in the mixed culture fermentation with *M. pulcherrima* involved or in the monoculture fermentation of *S. cerevisiae*, their trends initially increased and then decreased, but the time and magnitude of reaching the peak are different. During the mixed culture fermentation processes, *M. pulcherrima* declined significantly following *S. cerevisiae* inoculation, especially after 48 h, which could be due to the nutrient competition from *S. cerevisiae* and the inhibitory effects of its metabolites, and it might be caused by cell-to-cell contact (Englezos et al., 2019). Further research would be needed to illuminate the interaction of these two species. Furthermore, *M. pulcherrima* disappeared after 7 days, which was because the ethanol content accumulated through fermentation had reached or exceeded the ethanol tolerance of *M. pulcherrima* (>4 and <8%, v/v). *M. pulcherrima* disappeared in the middle and later stages of fermentation, while *S. cerevisiae* gradually became the sole species, this can also be explained by the competitive mechanism when different species inhabit the same niche (Tronchoni et al., 2017).

During the fermentation process, the inoculation of *M. pulcherrima* had a significant impact on the basic chemical composition of the icewine. Some existing studies have already confirmed that non-*Saccharomyces* yeasts can reduce ethanol level in wine fermentation (Contreras et al., 2014). Similarly, the decrease in ethanol level caused by adding *M. pulcherrima*, which is consistent with the result of Krêgiel et al. (2022) and Torres-Díaz et al. (2024); this may be due to the consumption of sugar by *M. pulcherrima* for production of glycerol or pyruvic acid. Reducing the ethanol content of wine is precisely in line with the current consumers’ considerations regarding the taste and health (Ballester-Tomás et al., 2017). On the contrary, the addition of *M. pulcherrima* had a reverse effect on the level of residual sugar. Moreover, the addition of *M. pulcherrima* lead to a decrease in the content of acetic acid, which is consistent with the results reported by Barbosa et al. (2018), and which were lower than the maximum allowable value of 2.1 g/L (International Organisation of Vine and Wine [OIV], 2026). *M. pulcherrima* can also increase the glycerol content, which is consistent with the results reported by Ruiz et al. (2018). Glycerol is one of the important products of yeast fermentation, and higher glycerol levels are considered to improve the quality of wine (Heit et al., 2018). The increase in glycerol production can be attributed to the overexpression of the GDP1 gene in *S. cerevisiae*; during the mixed culture fermentation process involving *M. pulcherrima*, the overexpression of this gene in *S. cerevisiae* was over induced (Mohand et al., 2017).

Subsequently, the impact of mixed culture of *M. pulcherrima* and *S. cerevisiae* strains during fermentation on the aroma profile of Vidal blanc icewine was focused on. In term of higher alcohols, they are produced by the deamination of amino acids which is caused by living yeast cells during fermentation to meet protein requirements, via the Ehrlich pathway (Belda et al., 2017). During the fermentation process, the addition of *M. pulcherrima* significantly increased the yields of isobutanol and 1-butanol, which is consistent with the results reported by Prior et al. (2019) and Torres-Díaz et al. (2024). Meanwhile, the addition of *M. pulcherrima* reduced the levels of phenylethanol and 1-propanol, which is in line with the research results of Zhang et al. (2018). Moreover, due to the strain-specificity within *S. cerevisiae* caused the differences in 2-methylbutan-1-ol, and the other higher alcohols (Li et al., 2022). The results reported by Krêgiel et al. (2022) demonstrated a significant increase in the level of 2-methylbutan-1-ol during the mixed fermentation of *S. cerevisiae* combined with *M. pulcherrima*, as compared to the monoculture fermentation of apple wine using *S. cerevisiae* alone.

Ester compounds tend to give icewine the desirable fruity and floral notes to icewine (Hong et al., 2021). *M. pulcherrima* can significantly increase production of isoamyl acetate and isobutyl acetate in icewine, and these two esters give icewine its floral and fruity aromas of bananas, apples, pears, etc., (Table 3). The increase in the production of isobutyl acetate is consistent with the results reported by Prior et al. (2019) and Torres-Díaz et al. (2024). Notably, the production of the precursor substance isobutanol has also increased. Moreover, *M. pulcherrima* could decrease propyl acetate and phenylethyl acetate in the icewines; similarly, this corresponds to the result that the levels of 1-propanol and phenylethyl alcohol are also relatively high in monoculture fermented icewine. Besides, *M. pulcherrima* could reduce the levels of apricolin, which is a lactone with a low odor detection threshold and is often detected in Baijiu (Wu et al., 2024). Ethyl esters are formed through enzyme-catalyzed condensation reactions between ethanol and acyl-CoA components (Saerens et al., 2010). In general, the low-fat ethyl esters exhibit a variety of fruit flavors (such as banana apple, pineapple, and strawberry), while the high-fat ethyl esters tend to have oily or fatty characteristics (Hong et al., 2021). In this study, *M. pulcherrima* was found to increase the yields of ethyl acetate, ethyl caprylate, ethyl caprate, ethyl nonanoate, ethyl hexanoate, which is consistent with the results reported by Krêgiel et al. (2022). Furthermore, as for the differences in the level of ethyl heptanoate in the different icewines, suggesting that *M. pulcherrima* had different regulatory mechanisms for ester expression (Varela and Borneman, 2017), and *S. cerevisiae* exhibited intraspecific differences in the expression in ethyl heptanoate of the mixed fermentation products (Hong et al., 2021).

Decanoic acid is the only acid that was detected, and it is naturally produced by yeast during the fermentation process, and intentionally adding it can terminate the fermentation process at the appropriate time (Baniţă et al., 2023). Moreover, *M. pulcherrima* could decrease 2, 4-dimethylbenzaldehyde production, which is beneficial to the aroma of the icewine. *M. pulcherrima* exhibit differential regulation of the expression of lauryl aldehyde, lauryl aldehyde can cause an unpleasant odors in wine when it is present in high concentrations (Liu et al., 2016). Besides, *M. pulcherrima* can also increase the yield of decanal. Decanal, which has the citrus flavor, has a significant impact on the overall wine aroma.

**FIGURE 3:**
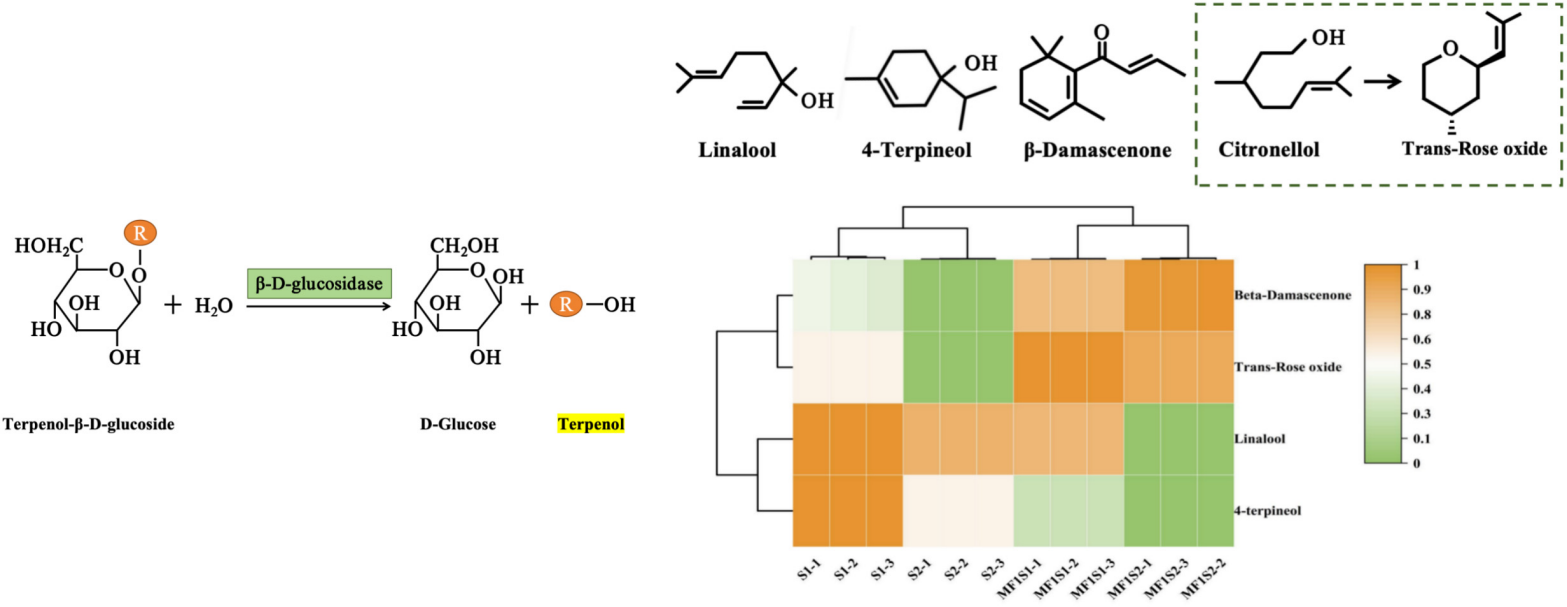
β-glucosidase involved in the metabolic reactions (from the hydrolysis of bound precursor substances to free terpenoid compounds).

In term of monoterpenols and C-13 norisoprenoid compounds, *M. pulcherrima* can increase the yields of trans-rose oxide and β-damascenone. Trans-rose oxide is a compound derived from the oxidation of citronellol, and it is an important aromatic compound that gives wine its green, lychee, and rose-like scents (Li et al., 2022). β-damascenone is a C13-norisoprenoid compound, which is the key aroma component of Vidal icewine; it is generally regarded as imparting pleasant floral and fruity scents and honey-like flavor to wine, and its sensory threshold is extremely low (only 0.05 μg/L), and even a slight change in its concentration can have a significant impact on the sensory evaluation of icewine (Zhang et al., 2018). It is worth noting that β-glucosidase has a significant impact on the yield of some metabolites, for instance, β-damascenone, linalool, 4-terpineol, and trans-rose oxide. Figure 3 shows the metabolic reactions of β-glucosidase during fermentation process, which produce monoterpenoid alcohols and C13-norisoprenoid compounds in this study. β-Glucosidase also affected the yields of phenethyl alcohol, and some higher alcohols (isoamylol and isobutanol). These higher alcohols are aroma substances, and are also precursors for the formation of more fruity esters. Further investigations should be conducted on the study of the interaction mechanism between *M. pulcherrima* and *S. cerevisiae*.

## 5 Conclusion

Metschnikowia pulcherrima has been studied and applied in winemaking, but its application in icewine fermentation is rarely reported. In this study, the indigenous *M. pulcherrima* strain with good tolerance performance and β-glucosidase activity was added to the fermentation of icewine. *M. pulcherrima* was completely replaced by *S. cerevisiae* in the middle and late stages of mixed culture fermentations of icewine. Compared with the monoculture fermentation icewine, the mixed-culture fermentation icewine has lower concentrations of acetic acid and ethanol, and higher concentration of glycerol. The addition of *M. pulcherrima* significantly increased the levels of several important volatile compounds, including β-damascenone, trans-rose oxide, isobutanol, isoamyl acetate, isobutyl acetate, and so on.

## Data Availability

The raw data supporting the conclusions of this article will be made available by the authors, without undue reservation.

## Footnotes

1
http://www.metaboanalyst.ca/
