# Supplementary figures and images for: Enological potential and application of Metschnikowia pulcherrima in Vidal blanc icewine fermentation
Source: Front Microbiol. 2026 Feb 25;17:1757951. doi: 10.3389/fmicb.2026.1757951 (PMC12975764; doi:10.3389/fmicb.2026.1757951)

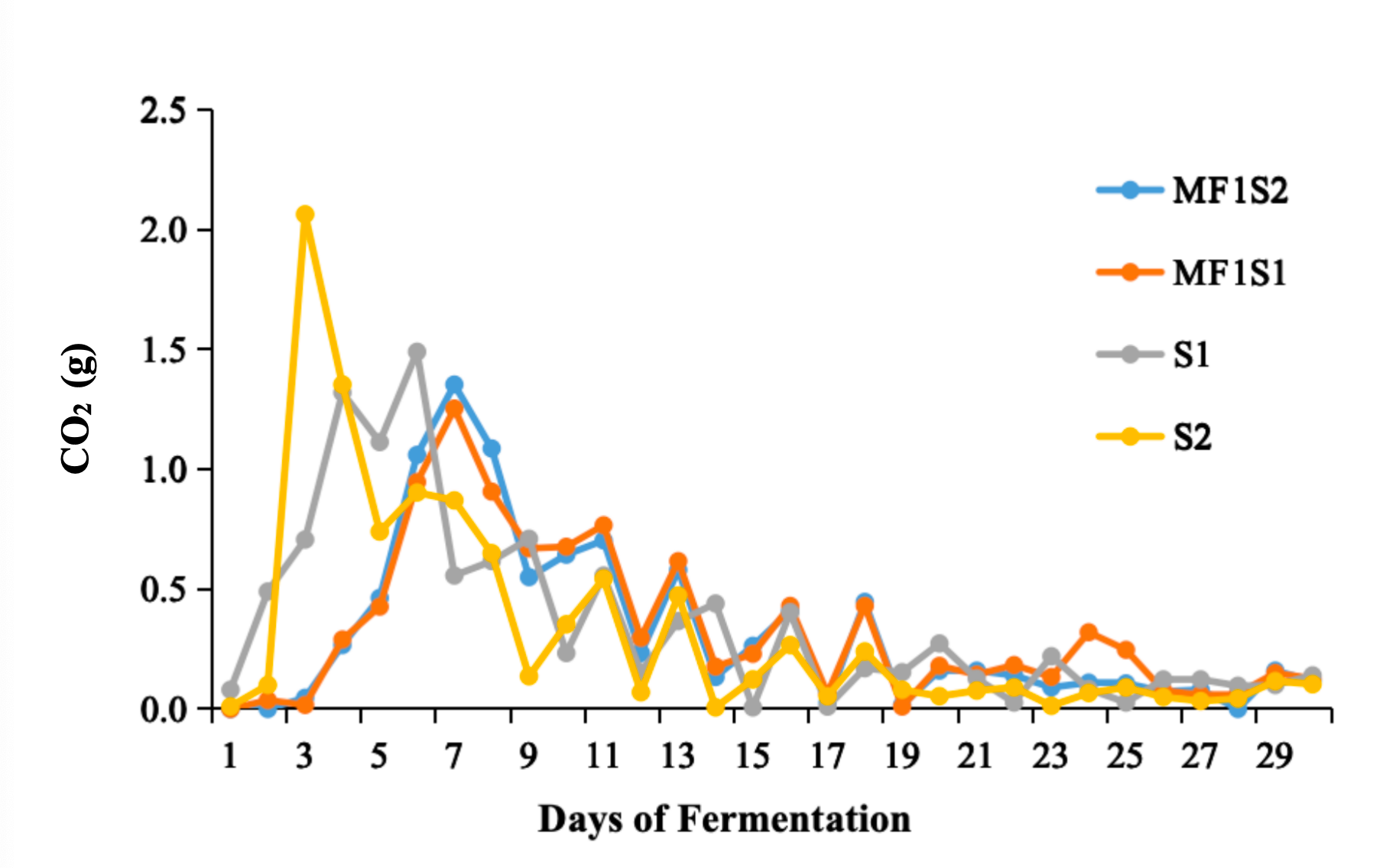

Supplement: Supplementary file 1 [file Image_1.tif]
